# Automatic Computer-Based Detection of Epileptic Seizures

**DOI:** 10.3389/fneur.2018.00639

**Published:** 2018-08-09

**Authors:** Christoph Baumgartner, Johannes P. Koren, Michaela Rothmayer

**Affiliations:** ^1^Department of Neurology, General Hospital Hietzing with Neurological Center Rosenhügel, Vienna, Austria; ^2^Karl Landsteiner Institute for Clinical Epilepsy Research and Cognitive Neurology, Vienna, Austria; ^3^Medical Faculty, Sigmund Freud University, Vienna, Austria

**Keywords:** seizure, detection, scalp-EEG, ECG, sEMG, SUDEP

## Abstract

Automatic computer-based seizure detection and warning devices are important for objective seizure documentation, for SUDEP prevention, to avoid seizure related injuries and social embarrassments as a consequence of seizures, and to develop on demand epilepsy therapies. Automatic seizure detection systems can be based on direct analysis of epileptiform discharges on scalp-EEG or intracranial EEG, on the detection of motor manifestations of epileptic seizures using surface electromyography (sEMG), accelerometry (ACM), video detection systems and mattress sensors and finally on the assessment of changes of physiologic parameters accompanying epileptic seizures measured by electrocardiography (ECG), respiratory monitors, pulse oximetry, surface temperature sensors, and electrodermal activity. Here we review automatic seizure detection based on scalp-EEG, ECG, and sEMG. Different seizure types affect preferentially different measurement parameters. While EEG changes accompany all types of seizures, sEMG and ACM are suitable mainly for detection of seizures with major motor manifestations. Therefore, seizure detection can be optimized by multimodal systems combining several measurement parameters. While most systems provide sensitivities over 70%, specificity expressed as false alarm rates still needs to be improved. Patients' acceptance and comfort of a specific device are of critical importance for its long-term application in a meaningful clinical way.

## Introduction

In general, automatic seizure detection must be distinguished from automatic seizure prediction. While seizure detection methods aim to detect ongoing seizures as soon as possible after seizure onset, seizure prediction models try to identify upcoming seizures well before seizure onset. In the present review we will focus on automatic seizure detection, while the reader is referred to excellent reviews on the current status of seizure prediction (1–4).

Automatic computer-based seizure detection currently is one of the major research questions in clinical epileptology for the following reasons:

1. Documentation of seizure frequency, seizure severity and seizure type by patients and their relatives represents the most important outcome parameter for epilepsy treatment both in everyday clinical practice and for the assessment of the efficacy of medical and non-medical treatment interventions in clinical trials. However, seizure documentation by patients and their relatives has been shown to be highly unreliable. In a study using video-EEG monitoring as the gold standard for seizure documentation, 55.5% of all seizures, 73.2% of complex partial seizures, 26.2% of simple partial seizures, 41.7% der secondary generalized tonic-clonic seizures, 85.8% of seizures arising out of sleep and 32.0% of seizures arising out of wakefulness remained unnoticed or were not documented by patients (5). In a study using a long-term, implanted seizure advisory system in patients with drug-resistant focal epilepsy for several months, a very low agreement between seizures noticed in patients' seizure diaries and those objectively documented on invasive EEG was found. Most patients significantly underestimated their seizure frequency. Because the relationship between patient and EEG documented seizures was highly variable from month to month, the application of a hypothetical correction factor seems not feasible (6). Therefore, objective and automatic documentation of seizure frequency, seizure severity, and seizure type is urgently needed in everyday clinical epileptology, but also in clinical epilepsy research to objectively assess the efficacy of therapeutic interventions in clinical trials (7).
2. Persons with active epilepsy face a standardized mortality ration of 2–3 (8, 9). Patients with severe severely drug-refractory epilepsy even suffer from a sevenfold increase in mortality rate over 3 years (10). The most frequent causes of death include cerebrovascular diseases, pneumonia, and neoplasia (8). Sudden unexpected death in epilepsy (SUDEP) represents the most frequent epilepsy associated cause of death and is most probably mediated through seizure associated cardiac, pulmonary or other autonomic dysfunctions (11). Automatic monitoring of EEG, cardiac, respiratory, and other autonomic changes during seizures therefore could be useful to elucidate the pathomechanisms underlying SUDEP (12). Generalized tonic-clonic seizures occurring out of sleep are a significant risk factor for SUDEP with supervision during night currently representing the only preventive measure (11, 13, 14). Automatic detection of nocturnal generalized tonic-clonic seizures could alert relatives, friends or caregivers leading to check on the patient, and provide sufficient stimulation to prevent respiratory arrest (15).
3. Automatic seizure detection systems could be used as seizure alarm devices for patients without reliable auras alleviating the unpredictability of seizures and their potential social embarrassments. Automatic seizure detection systems and warning devices could also help to prevent seizure associated injuries (10). This would significantly reduce the fear of seizures and thus improve the quality of life for persons with epilepsy (16). However, warning devices based on seizure detection would be clinically useful only in patients with subclinical seizures detected by the system or in order to alert significant others about an ongoing seizure enabling them to set protective measures. On the contrary, a warning device would be useless for patients if seizures have already started and lead to impairment of consciousness. Therefore, warning devices ideally should be based on seizure prediction rather than on seizure detection.
4. Automatic seizure detection systems could open the way to on-demand therapies, such as acute administration of anticonvulsants or acute electrical stimulation in selected brain areas, in order to stop ongoing seizures (7, 17–21).
5. During video-EEG-monitoring (VEM) in the epilepsy monitoring unit (EMU), applications for automatic seizure detection systems include enhancement of patient safety, more efficient data analysis, automatic documentation of seizures, and computer-based neurological and neuropsychological testing during and after seizures.
  Patient safety in order to avoid seizure related injuries, to recognize seizure induced cardiac arrhythmias and finally to prevent SUDEP has become a central issue during VEM (22–25). While optimum patient safety can be ensured only by continuous observation through trained personnel (25, 26), several surveys showed that only 56–80% of EMUs can provide continuous personal patient surveillance (23–25, 27). Automatic on-line seizure detection and warning systems could provide a significantly less personnel intensive alternative to personal patient surveillance in the EMU. However, only 15–19% of EMUs actually use on-line seizure detection and warning systems (24, 27).
  Automatic seizure detection could optimize data review in the EMU and thus could facilitate a more efficient personnel assignment (28–30). Although agreement on EEG seizure identification between human electroencephalographers is high with an average any-overlap sensitivity of 92% and false positives per hour rate of 0.117 applying any-overlap comparisons (i.e., whether there was any detection overlap between experts during a period annotated as a seizure), high seizure rates as well as short and long seizure durations with ambiguous offsets can make analysis rather complicated resulting in suboptimal agreements even between EEG experts (31). Therefore, automatic seizure detection systems could improve seizure documentation during VEM.
  The exact analysis of clinical seizure semiology is essential for correct seizure classification as well as for the localization of the seizure onset zone and pathways of seizure spread (32, 33). Proper assessment of many essential features of seizure semiology require systematic interactive testing for various cognitive, behavioral, sensory, and motor functions during and after seizures (32, 33). However, immediate ictal testing frequently is not possible due to personnel limitations (34). Recently, a seizure detection system triggering automatically a series of video-recorded behavioral tasks presented in the patient's room [Automatic Responsiveness Testing in Epilepsy (ARTiE)] has been introduced which could optimize assessment of these functions during seizures (34).
6. Finally, automatic seizure detection has become increasingly important for the detection of non-convulsive seizures and non-convulsive status epilepticus in critical care patients (35).

## Performance measures of automatic seizure detection and alarm algorithms

Visual annotation of seizures by EEG experts remains the gold standard for performance evaluation of seizure detection and seizure alarm algorithms. In general, agreement on seizure annotation between EEG experts is high. However, discrepancies can occur in case of high seizure rates as well as in case of seizures with short and long durations. These caveats have to be considered during assessment of computer-based seizure detection algorithms (31). Preferably, agreement on seizure annotations should be obtained by several blinded EEG experts (29, 36).

Seizure detection and alarm algorithms usually are evaluated and compared concerning the following performance measures (1, 37):

1. True positives (TP) meaning that the algorithm detected a seizure identified by the human expert;
2. False negatives (FN) meaning that the algorithm missed a seizure identified by the human expert;
3. False positives (FP) meaning the algorithm erroneously detected a seizure which was not confirmed by the human expert;
4. Sensitivity defined as the ratio TP/(TP + FN);
5. Specificity defined as the number of false positive alarms per hour referred to as false positive alarm rate (FAR).
6. Alarm algorithms need to provide both on-line calculation and short detection delays.
7. Detection delay is defined as the time interval between the time of seizure onset identified by the human expert and the time when the computer algorithm sets the alarm. Detection delays in the range of a few seconds are required for alarm devices (38, 39).

## Measurement parameters for automatic seizure detection

Ictal EEG changes which can be measured on scalp-EEG and intracranial EEG represent the electrophysiological correlates of epileptic seizures. Therefore, analysis of ictal EEG represents the most direct biomarker for automatic epileptic seizure detection. While specificity of intracranial EEG is considerably higher than that of scalp-EEG, drawbacks of intracranial EEG based seizure detection include the sampling problem due to limited coverage of the cerebral cortex and a less well defined specificity as compared to scalp-EEG. Motor manifestations representing a prominent feature of many seizures can be assessed with surface electromyography (sEMG), accelerometry (ACM), video detection systems, and mattress sensors. Most epileptic seizures are accompanied by changes in physiologic parameters like heart and respiration rate, oxygen saturation, skin temperature, and sweat secretion. These parameters can be measured by electrocardiography (ECG), respiratory monitors, pulse oximetry, surface temperature sensors, and electrodermal activity (EDA) (Table 1).

**Table 1:** Measurement parameters for automatic seizure detection.

**Electrophysiological correlates of epileptic seizures**
Scalp-EEG
Intracranial EEG
**Measurement of motor manifestations**
Surface electromyography (semg)
Accelerometry (ACM)
Video detection systems
Mattress sensors
**Measurement of physiologic parameters**
Heart rate → electrocardiography (ECG)
Respiration rate → respiratory monitors
Oxygen saturation → pulse oximetry
Skin temperature → surface temperature sensors
Sweat secretion → electrodermal activity (EDA)

We focused our review on automatic seizure detection based on scalp electroencephalography (scalp-EEG), electrocardiography (ECG) and surface electromyography (sEMG) because these modalities have been studied most extensively in the literature. For seizure detection based on other modalities the reader is referred to some recent excellent reviews (12, 16, 40).

## Automatic seizure detection based on scalp electroencephalography (scalp-EEG)

Ideally scalp-EEG based seizure detection algorithms should detect a broad range of seizures in patients with different epilepsy syndromes and seizure-onset zones with high sensitivity and specificity. Algorithms should facilitate fast and robust analysis of large amounts of EEG data. All EEG data acquired during VEM should be analyzed, including artifacts, all neurophysiological states as well as non-ictal physiological and pathological EEG patterns (37).

While scalp-EEG based seizure detection algorithms can use either single or multiple scalp-EEG channels, most algorithms applied in a clinical setting use multiple EEG channels. In multiple channel systems, the montage can significantly influence for performance of the algorithm (41). For computational reasons and for patient comfort (especially in an outpatient setting) a proper selection and reduction of electrode numbers is important (42–44). While using all 21 channels of the International 10-20-system provided a sensitivity of 84%, reduction to only 8 frontal and temporal electrodes yielded an average sensitivity of 79%, a restriction to only 7 temporal, parietal, and occipital electrodes an average sensitivity of 68% (43). After data acquisition, artifact rejection needs to be applied. Detection of EEG seizure patterns is based on characteristic changes with respect to frequency, amplitude and/or rhythmicity using different linear and non-linear time-frequency signal analyses techniques (37, 45, 46).

In general non-patient specific and patient specific algorithms can be distinguished. Non-patient specific algorithms can be applied without a priori knowledge about the patient's individual electrographic seizure patterns. Therefore these algorithms are easy to use with identical parameter settings for all patients which is important for the application in a busy clinical environment. Patient specific algorithms, on the other hand, try to improve the performance by parameter adjustments for each individual patient (47–49). However, such patient specific detection algorithms need specific training and interactive, sometimes complex individualized parameter adjustments.

Table 2 provides a selection of non-patient specific algorithms tested in a clinical setting (28–30, 37–39, 41, 46, 50–58).

**Table 2:** Non-patient specific, scalp-EEG based seizure detection algorithms tested in clinical settings.

**References**	**EEG sample (hours)**	**Patients**	**Seizures**	**Sensitivity (%)**	**Specificity FAR (per hour)**	**Detection delay (seconds)**
Gotman (50)	4362	44	179	73.2	0.84	n.a.
Pauri et al. (28)	461	12	253	81.4	5.38	n.a.
Gabor et al. (51)^+^	528	22	62	90.3	0.71	n.a.
Gabor (52)^*^	4554	65	181	92.8	1.35	n.a.
Wilson et al. (53)	1049	426	672	76.0	0.11	n.a.
Saab and Gotman (38)	360	16	69	76.0	0.34	10.0
Kuhlmann et al. (54)	525	21	88	81.0	0.60	16.9
Meier et al. (38)	1403	57	91	> 96.0	<0.5	2.0
Schad et al. (55)	423	6	26	59	0.15	n.a.
Kelly et al. (29)	1200	55	146	79.5	0.08	n.a.
Zandi et al. (46)	236	26	79	91.0	0.33	7.0
Hopfengärtner et al. (41)	3248	19	148	90.9	0.29	19
Hopfengärtner et al. (37)	25278	159	794	87.3	0.22	n.a.
Hartmann et al. (56)	4300	48	186	83.0	0.3	n.a.
Fürbass et al. (57)	22000	275	623	73.0	0.30	n.a.
Fürbass et al. (30)^*^	15684	205	526	81.0	0.29	n.a
Fürbass et al. (30)^+^	25567	310	113	75.0	0.30	n.a.

*FAR, false positive alarms per hour*;

+ *development data set*;

* *prospective data set*.

Due to the large amount of EEG data, patients and seizures analyzed we summarize here the studies published by the Erlangen group (37, 41) and the Vienna group (30, 56, 57).

Hopfengärtner et al. (37, 41) analyzed 25,278 h of EEG in 159 patients [117 temporal-lobe epilepsies (TLE); 35 extra-temporal lobe epilepsies (ETLE); 7 others] containing 794 seizures. They applied an adaptive thresholding technique and calculated the integrated power in the frequency band from 2.5 to 12 Hz for a seizure detection montage including basal temporal electrodes referenced against the average of Fz-Cz-Pz. With this approach they could obtain a sensitivity of 87.3% and a FAR of 0.22/h. Performance for TLE patients (18,996 h of EEG including 589 seizures; sensitivity 89.9%, FAR 0.19/h) was better as compared to ETLE patients (5,192 h of EEG including 172 seizures; sensitivity 77.4%, FAR 0.25/h).

Fürbass et al. (30) performed a prospective multi-center study in 3 epilepsy centers. The algorithm was developed on additional 25,567 h of EEG from 310 patients (including 124 patients with 1,113 seizures and an additional 186 patients without seizures) (30, 56, 57). While for the prospective data set a mean sensitivity of 81% and a FAR 0.29/h could be obtained, in the development dataset mean sensitivity was 75% and FAR was 0.3/h. In the prospective data set, 16 seizures unnoticed during routine visual analysis (3% of all seizures) were detected by the algorithm. Sensitivity was better for TLE (83%) than for ETLE (64%).

Table 3 summarizes some clinically applied patient specific algorithms (47–49, 58–61). Of course performance is higher for patient-specific as compared to non-patient specific algorithms. Nevertheless it should be mentioned the amount of EEG, patients and seizures reported in the literatures is drastically higher for non-patient specific algorithms indicating their easier use in clinical setting.

**Table 3:** Patient specific, scalp-EEG based seizure detection algorithms tested in clinical settings.

**References**	**EEG sample (hours)**	**Patients**	**Seizures**	**Sensitivity (%)**	**Specificity FAR (per hour)**	**Detection delay (seconds)**
Qu and Gotman (47)	1071	10	n.a.	n.a.	1.40	n.a.
Qu and Gotman (59)	29.7	12	35	100	0.03	9.5
Qu and Gotman (60)	n.a.	12	47	100	0.02	9.35
Shoeb et al. (61)	60	36	139	94	0.25	8.0
Khamis et al. (48)	1624	10	83	91.6	0.27	n.a.
Minasyan et al. (49)	625	25	86	100	0.02	4.0

*FAR, false positive alarms per hour*.

In conclusion, non-patient specific scalp-EEG based seizure detection algorithms provide sensitivities between 73 and 96% (62). Difficult to detect are EEG seizure patterns with short duration, with low amplitude, with circumscribed highly focal activity, with high frequency, with unusual non-rhythmic morphology and those obscured by artifact. These features frequently apply to seizures of extratemporal origin which therefore are more difficult to detect than seizures of temporal lobe origin (30, 37, 58). Specificity (FAR) of non-patient specific scalp-EEG based seizure detection algorithms varies between 0.11 and 5.38/h. Low FARs are essential for the acceptance of an algorithm in a clinical setting, especially if the algorithm is applied as an alarm device. Here high FAR would result in unnecessary concerns and anxiety of patients as well as frequent unnecessary responses and actions by caregivers. FAR can be caused by physiological and pathological brain activity including sleep patterns, rhythmic non-epileptiform activities like frontal intermittent rhythmic delta activity (FIRDA) or temporal intermittent rhythmic delta activity (TIRDA) or by various especially rhythmic artifacts (including chewing, tooth brushing, repetitive movements, eye movements etc.) (30, 37, 58). Algorithms need to be developed and tested on large amount of EEG data containing also prolonged time periods of interictal EEG across all stages of the sleep-waking cycle and including all kinds of artifacts and non-ictal physiological and pathological EEG patterns in order to obtain stable and reproducible results in a clinical setting (30, 37, 58).

While patient specific algorithms can further enhance sensitivity and selectivity, drawbacks of these approaches include sometimes complex parameter adjustments and the necessity of training for individual patients (47–49).

If a detection algorithm is used as an alarm system, on-line calculation with short detection delays represents a prerequisite. Detection delays reported in the literature vary between 2 and 19 s (39).

Draw-backs of scalp-EEG based seizure detection systems include the complexity of the EEG signal, attenuation of the EEG signal by skull and scalp and the fact that large parts of the cerebral cortex including mesial frontal, basal frontal, and mesial temporal areas are not accessible to the scalp-EEG. The most significant limitation remains the application of scalp-EEG based seizure detection systems in an outpatient setting because it is not acceptable for patients to wear EEG electrode arrays for prolonged time periods in everyday life (58). Recently developed subcutaneous EEG electrodes may offer a practical solution for this problem (63). Chronically implanted intracranial electrodes represent another option for long-term outpatient EEG recordings which have been successfully applied for seizure prediction (6) and seizure detection with responsive brain stimulation (17–21). However, despite their invasive nature these devices suffers from high rates of false positive detections limiting the clinical usefulness for seizure detection in a clinical setting (64). The reader is referred to an recent excellent paper on the problems and future aspects of seizure detection based on invasive EEG recordings (64).

## Automatic seizure detection based on electrocardiography (ECG)

ECG represents a simple and easy to record signal for automatic seizure detection. Many seizures are accompanied by a pronounced ictal sinus tachycardia (65). Ictal sinus tachycardia is caused primarily by direct activation of the central autonomic network through epileptic discharges and to a much lesser extent the mere consequence of motor manifestations during epileptic seizures (66). Cortical areas of the central autonomic network include the amygdala, the anterior insula, the anterior cingulate cortex, the ventromedial prefrontal cortex, and the posterior orbitofrontal cortex. Subcortical areas of the central autonomic network are represented in the hypothalamus, the periaqueductal gray matter, the parabrachial Köller-Fuse region, the nucleus of the tractus solitarius, the nucleus ambiguous and the ventrolateral medulla oblongata (67).

Compared to the EEG, the ECG signal is highly robust and less prone to artifacts. Long-term ECG recordings can be easily obtained in an ambulatory setting using ambulatory ECG, smart watches and minimally invasive implantable loop recorders for prolonged time periods. Contrary to long term scalp EEG recordings these systems impose no burdens or restrictions to the patient and are well tolerated by patients. Compared to long-term intracranial EEG recordings, implantable loop recorders are far less invasive, carry only negligible risks for the patients, are widely available commercially and considerably less costly than implantable EEG recording devices. Finally, the ECG signal is simpler to process and to analyze than the EEG signal.

Definition of ictal sinus tachycardia is rather heterogeneous in the literature (65). The most frequent definition refers to a heart rate >100 beats per minute (bpm) corresponding to the threshold for a maximum normal heart rate for patients older than 15 years. Other definitions include a heart rate >120 bpm, a heart rate >10 bpm above baseline heart rate, age-adjusted thresholds and not further specified significant changes in heart rate relative to baseline (65).

In general, a lower threshold will result in a higher sensitivity, but lower specificity corresponding to a higher FAR, whereas a higher threshold will be associated with a lower sensitivity, but a higher specificity corresponding to a lower FAR.

Concerning algorithms for detection of ictal sinus tachycardia, so-called threshold and curve fitting algorithms can be distinguished. Threshold algorithms set an alarm when the average heart frequency in an analyzing time window exceeds the average heart frequency in a baseline time window by a predefined threshold parameter of 2.5–25 bpm. The duration of the analyzing time window can be varied for instance from 5 to 15 s, while the duration of the baseline time window is kept constant at 20 s (68). The most recent version of the vagal nerve stimulator incorporates a so-called cardiac based seizure detection algorithm (CBSDA) which compares the most recent heart rate to a background heart rate established over approximately the previous 5 min of R–R intervals. Whenever heart rate (during a presumed seizure) exceeds baseline heart by 20–70% (the actual value can be programmed in 10% increments) for at least 1 s an automatic on-demand stimulation is triggered in a closed loop fashion (69). Curve fitting algorithms on the contrary calculate changes in heart frequency based on predefined algorithms resulting in an increased specificity and shortened detection latency (68, 70, 71).

According to a recent review article incorporating 34 articles ictal sinus tachycardia can be observed in 82% of patients (65). While some studies reported consistent ictal heart rate changes within a given patient (72, 73), others observed intraindividual variability of ictal heart rate changes (68, 71, 74–76). The absolute increase in heart rate averaged 34.23 bpm per seizure and 33.51 bpm per patient (weighted average across several studies) (65). Concerning seizure types, ictal sinus tachycardia was observed in 12% of subclinical seizures, in 71% of focal onset seizures, in 64% of generalized seizures and in 76% of mixed seizure types (weighted average across several studies) (65). Concerning seizure onset zone, ictal sinus tachycardia was more consistent and prevalent in seizures of temporal origin as compared to those of extratemporal origin (65). While the effect of seizure onset lateralization was inconsistent (65), some studies suggest a more pronounced increase in heart rate during seizures arising from the non-dominant hemisphere (70, 71, 77–79). Secondarily generalized tonic-clonic seizures result in a higher ictal heart rate as compared to complex partial seizures (80). Finally, an elevated heart rate was observed already prior to seizure onset in those focal seizures evolving to secondarily generalized seizures as compared to those focal seizures which remained localized (81).

In a study with intracranial electrodes, the temporal relationship between the onset of ictal tachycardia, seizure onset on intracranial EEG, seizure onset on scalp-EEG and clinical seizure onset was investigated (82). Ictal tachycardia occurred after seizure onset on intracranial EEG in all seizures with mean latencies of 21.6–23.7 s. On the contrary, ictal tachycardia preceded scalp-EEG onset in 9/13 patients and in 48/78 seizures with mean latencies of 7.8–14.0 s. Furthermore, ictal tachycardia occurred before the first clinical sign in 10/13 patients and in 56/78 seizures with mean latencies of 6.5–9.5 s. Ictal tachycardia was observed earlier in seizures arising from the hippocampal formation than in those of extrahippocampal onset. Finally, ictal tachycardia occurred earlier in seizures originating from the right temporal lobe as compared to those originating from the left temporal lobe. The authors concluded that ictal tachycardia is an ictal rather than a preictal phenomenon and that ictal tachycardia may be an appropriate noninvasive marker for closed-loop interventions (82).

Concerning specificty, heart rate increases during epileptic seizures occur faster and are more pronounced as compared to those associated with physical exercise or nocturnal arousals (83, 84). In the VNS study, a mean sensitivity of 80% with a FAR of 0.5–7.2/h and a detection latency of 6–35 s could be achieved using the cardiac based seizure detection algorithm (CBSDA) (69).

Analysis of heart rate variability represents a very promising approach for ECG based seizure detection. Especially the so-called modified cardiac sympathetic index (CSI100) seems to be suitable to detect the abnormal increase in sympathetic tone during epileptic seizures (85). Thus, the CSI100 based algorithm showed an excellent performance for seizure detection: All seizures were detected in 13/17 patients with a mean detection latency of 16 s (range: 6 s before and 50 s after EEG or clinical seizure onset). Furthermore, the CSI100 based algorithm could differentiate very well between ictal ECG changes and physiologic, exercise-induced ECG changes, while a simple analysis of the heart rate failed to do so (85).

## Automatic seizure detection based on surface electromyography (sEMG)

Quantitative analysis of surface electromyography (sEMG) represents a valuable tool for the detection of seizures with prominent motor manifestations (16).

The deltoid muscle, the anterior tibialis muscle (86–89) as well as the brachial biceps and triceps muscles (90, 91) have been used as recording sites for sEMG based seizure detection.

For the detection of generalized tonic-clonic seizures sEMG from the deltoid muscle yielded higher sensitivity, but lower specificity than sEMG from the anterior tibialis muscle [deltoid muscle: sensitivity 100%, false positive alarm rate 1 per 24 h, detection latency 13.7 s (86); anterior tibialis muscle: sensitivity 57%, false positive alarm rate 1 per 12 days, detection latency 25 s (87)]. For the detection of tonic seizures with sEMG applied to the deltoid muscle a sensitivity of 53–63% with a false positive alarm rate of 0.08 bis 7.90/h could be obtained (89).

Continuous sEMG recordings for 1,399 h from the brachial biceps and triceps muscle were performed in 33 patients with 196 epileptic seizures (21 generalized tonic-clonic seizures, 96 myoclonic, 28 tonic, 12 absence, and 42 focal seizures with or without loss of awareness) and 4 nonepileptic spells. The algorithm detected 20 of 21 generalized tonic-clonic seizures corresponding to a sensitivity of 95% with an average detection latency of 20 s. While only one false positive alarm was observed in the postictal phase after a generalized tonic-clonic seizure, no false positive alarms were triggered by other seizure types (90).

In a prospective multicenter phase III trial in 199 patients investigated in 11 epilepsy monitoring units, sEMG from the brachial biceps muscle detected 35 out of 46 of generalized tonic-clonic seizures corresponding to a sensitivity of 76% with a false positive alarm rate of 2.52/24 h. If the device was correctly placed over the midline of the biceps muscle, 29/29 of generalized tonic-clonic seizures could be detected (sensitivity 100%, mean false positive alarm rate 1.44/24 h, mean detection latency 7.70 s). While mild to moderate adverse events (mostly skin irritation caused by the electrode patch that resolved without treatment) occurred in 28% of participants leading to study withdrawal in 9%, no serious adverse events were reported (91).

In a prospective study in 71 patients from 3 centers sEMG recordings from brachial biceps muscle (mean recording time per patient 53.19 h, total recording time 3735.5 h), a sensitivity of 93.8% (30 out of 32 generalized tonic-clonic seizures) with a median detection latency of 9 s (range: −4 to 48 s) and a false alarm rate was 0.67/24 h could be obtained. No adverse events were observed (92).

Quantitative sEMG analysis provides direct information about the electric activity in the motor cortex and therefore is useful to elucidate the pathomechanisms of convulsive seizures (93). Thus sEMG can differentiate between generalized tonic-clonic seizures and maximal voluntary muscle contraction (93). sEMG correctly classified 24/25 (96%) of generalized tonic-clonic seizures and 18/19 (95%) of psychogenic non-epileptic seizures corresponding to an overall diagnostic accuracy of 95% (94). Furthermore, the tonic phase of generalized tonic-clonic seizures showed different quantitative features as compared to tonic seizures. Furthermore, due to its high temporal resolution sEMG facilitates a detailed characterization of the temporal evolution of generalized tonic-clonic seizures suggesting that the same inhibitory mechanisms involved in the prevention of buildup of seizure activity, contribute to seizure termination. Thus, quantitative sEMG can be viewed as a neurophysiologic biomarker for detection of generalized tonic-clonic seizures and for the automated differentiation between convulsive and non-convulsive epileptic seizures (93).

## Conclusion

Automatic computer-based seizure detection and warning devices are important for objective seizure documentation, for SUDEP prevention, to avoid seizure related injuries and social embarrassments as a consequence of seizures, and to develop on demand epilepsy therapies. Automatic seizure detection systems can be based on direct analysis of epileptiform discharges on scalp-EEG or intracranial EEG, on detection of motor manifestations of epileptic seizures using surface electromyography (sEMG), accelerometry (ACM), video detection systems, and mattress sensors and finally on the assessment of changes of physiologic parameters accompanying epileptic seizures measured by electrocardiography (ECG), respiratory monitors, pulse oximetry, surface temperature sensors, and electrodermal activity (EDA). Different seizure types affect preferentially different measurement parameters. While EEG changes accompany all types of seizures, sEMG and ACM are suitable primarily for the detection of seizures with major motor manifestations. Therefore, multimodal systems combining several different measurement parameters certainly represent the future of automatic seizure detection (16, 58). While most systems provide sensitivities over 70%, specificity expressed as false alarm rates still needs to be improved. Patients' acceptance and comfort of a specific device are of critical importance for its long-term application in meaningful clinical way.

## Author contributions

CB conceived, designed and wrote the manuscript, prepared the tables and reviewed the final version. JK revised the manuscript, prepared the tables and proofread the final version. MR revised the manuscript, prepared the tables and proofread the final version of the paper.

### Conflict of interest statement

The authors declare that the research was conducted in the absence of any commercial or financial relationships that could be construed as a potential conflict of interest.

## References

[1] Mormann F Andrzejak RG Elger CE Lehnertz K. Seizure prediction: the long and winding road. Brain (2007) 130(Pt 2):314–33. 10.1093/brain/awl241 17008335

[2] Andrzejak RG Chicharro D Elger CE Mormann F. Seizure prediction: any better than chance? Clin Neurophysiol. (2009) 120:1465–78. 10.1016/j.clinph.2009.05.019 19576849

[3] Freestone DR Karoly PJ Peterson AD Kuhlmann L Lai A Goodarzy F Seizure prediction: science fiction or soon to become reality? Curr Neurol Neurosci Rep. (2015) 15:73 10.1007/s11910-015-0596-3 26404726

[4] Gadhoumi K Lina JM Mormann F Gotman J. Seizure prediction for therapeutic devices: a review. J Neurosci Methods (2016) 260:270–82. 10.1016/j.jneumeth.2015.06.010 26099549

[5] Hoppe C Poepel A Elger CE. Epilepsy: accuracy of patient seizure counts. Arch Neurol. (2007) 64:1595–9. 10.1001/archneur.64.11.1595 17998441

[6] Cook MJ O'Brien TJ Berkovic SF Murphy M Morokoff A Fabinyi G. Prediction of seizure likelihood with a long-term, implanted seizure advisory system in patients with drug-resistant epilepsy: a first-in-man study. Lancet Neurol. (2013) 12:563–71. 10.1016/s1474-4422(13)70075-9 23642342

[7] Elger CE Mormann F. Seizure prediction and documentation–two important problems. Lancet Neurol. (2013) 12:531–2. 10.1016/s1474-4422(13)70092-9 23642341

[8] Neligan A Bell GS Johnson AL Goodridge DM Shorvon SD Sander JW. The long-term risk of premature mortality in people with epilepsy. Brain (2011) 134(Pt 2):388–95. 10.1093/brain/awq378 21278406

[9] Nevalainen O Ansakorpi H Simola M Raitanen J Isojarvi J Artama M. Epilepsy-related clinical characteristics and mortality: a systematic review and meta-analysis. Neurology (2014) 83:1968–77. 10.1212/wnl.0000000000001005 25339211

[10] Strzelczyk A Griebel C Lux W Rosenow F Reese JP. The burden of severely drug-refractory epilepsy: a comparative longitudinal evaluation of mortality, morbidity, resource use, and cost using german health insurance data. Front Neurol. (2017) 8:712. 10.3389/fneur.2017.00712 29312132 PMC5743903

[11] Surges R Thijs RD Tan HL Sander JW. Sudden unexpected death in epilepsy: risk factors and potential pathomechanisms. Nat Rev Neurol. (2009) 5:492–504. 10.1038/nrneurol.2009.118 19668244

[12] Van de Vel A Cuppens K Bonroy B Milosevic M Jansen K Van Huffel S. Non-EEG seizure detection systems and potential SUDEP prevention: state of the art: Review and update. Seizure (2016) 41:141–53. 10.1016/j.seizure.2016.07.012 27567266

[13] Hesdorffer DC Tomson T Benn E Sander JW Nilsson L Langan Y. Combined analysis of risk factors for SUDEP. Epilepsia (2011) 52:1150–9. 10.1111/j.1528-1167.2010.02952.x 21671925

[14] Hesdorffer DC Tomson T Benn E Sander JW Nilsson L Langan Y. Do antiepileptic drugs or generalized tonic-clonic seizure frequency increase SUDEP risk? A combined analysis. Epilepsia (2012) 53:249–52. 10.1111/j.1528-1167.2011.03354.x 22191685

[15] Harden C Tomson T Gloss D Buchhalter J Cross JH Donner E. Practice guideline summary: sudden unexpected death in epilepsy incidence rates and risk factors: Report of the Guideline Development, Dissemination, and Implementation Subcommittee of the American Academy of Neurology and the American Epilepsy Society. Neurology (2017) 88:1674–80. 10.1212/wnl.0000000000003685 28438841

[16] Ulate-Campos A Coughlin F Gainza-Lein M Fernandez IS Pearl PL Loddenkemper T. Automated seizure detection systems and their effectiveness for each type of seizure. Seizure (2016) 40:88–101. 10.1016/j.seizure.2016.06.008 27376911

[17] Morrell MJ. Responsive cortical stimulation for the treatment of medically intractable partial epilepsy. Neurology (2011) 77:1295–304. 10.1212/WNL.0b013e3182302056 21917777

[18] Bergey GK Morrell MJ Mizrahi EM Goldman A King-Stephens D Nair D. Long-term treatment with responsive brain stimulation in adults with refractory partial seizures. Neurology (2015) 84:810–7. 10.1212/wnl.0000000000001280 25616485 PMC4339127

[19] Sun FT Morrell MJ. The RNS system: responsive cortical stimulation for the treatment of refractory partial epilepsy. Expert Rev Med Devices (2014) 11:563–72. 10.1586/17434440.2014.947274 25141960

[20] Geller EB Skarpaas TL Gross RE Goodman RR Barkley GL Bazil CW. Brain-responsive neurostimulation in patients with medically intractable mesial temporal lobe epilepsy. Epilepsia (2017) 58:994–1004. 10.1111/epi.13740 28398014

[21] Jobst BC Kapur R Barkley GL Bazil CW Berg MJ Bergey GK. Brain-responsive neurostimulation in patients with medically intractable seizures arising from eloquent and other neocortical areas. Epilepsia (2017) 58:1005–14. 10.1111/epi.13739 28387951

[22] Rheims S Ryvlin P. Patients' safety in the epilepsy monitoring unit: time for revising practices. Curr Opin Neurol. (2014) 27:213–8. 10.1097/wco.0000000000000076 24553465

[23] Shafer PO Buelow J Ficker DM Pugh MJ Kanner AM Dean P. Risk of adverse events on epilepsy monitoring units: a survey of epilepsy professionals. Epilepsy Behav. (2011) 20:502–5. 10.1016/j.yebeh.2010.12.048 21306957

[24] Rubboli G Beniczky S Claus S Canevini MP Kahane P Stefan H. A European survey on current practices in epilepsy monitoring units and implications for patients' safety. Epilepsy Behav. (2015) 44:179–84. 10.1016/j.yebeh.2015.02.004 25725329

[25] Hamandi K Beniczky S Diehl B Kandler RH Pressler RM Sen A. Current practice and recommendations in UK epilepsy monitoring units. Report of a national survey and workshop. Seizure (2017) 50:92–8. 10.1016/j.seizure.2017.06.015 28644984

[26] Rosenow F Bast T Czech T Feucht M Hans VH Helmstaedter C. Revised version of quality guidelines for presurgical epilepsy evaluation and surgical epilepsy therapy issued by the Austrian, German, and Swiss working group on presurgical epilepsy diagnosis and operative epilepsy treatment. Epilepsia (2016) 57:1215–20. 10.1111/epi.13449 27354263

[27] Kobulashvili T Hofler J Dobesberger J Ernst F Ryvlin P Cross JH. Current practices in long-term video-EEG monitoring services: a survey among partners of the E-PILEPSY pilot network of reference for refractory epilepsy and epilepsy surgery. Seizure (2016) 38:38–45. 10.1016/j.seizure.2016.03.009 27104922

[28] Pauri F Pierelli F Chatrian GE Erdly WW. Long-term EEG-video-audio monitoring: computer detection of focal EEG seizure patterns. Electroencephalogr Clin Neurophysiol. (1992) 82:1–9. 1370137 10.1016/0013-4694(92)90175-h

[29] Kelly KM Shiau DS Kern RT Chien JH Yang MC Yandora KA. Assessment of a scalp EEG-based automated seizure detection system. Clin Neurophysiol. (2010) 121:1832–43. 10.1016/j.clinph.2010.04.016 20471311 PMC2934863

[30] Furbass F Ossenblok P Hartmann M Perko H Skupch AM Lindinger G. Prospective multi-center study of an automatic online seizure detection system for epilepsy monitoring units. Clin Neurophysiol. (2015) 126:1124–31. 10.1016/j.clinph.2014.09.023 25454341

[31] Wilson SB Scheuer ML Plummer C Young B Pacia S. Seizure detection: correlation of human experts. Clin Neurophysiol. (2003) 114:2156–64. 10.1016/S1388-2457(03)00212-8 14580614

[32] Blume WT Luders HO Mizrahi E Tassinari C van Emde Boas W Engel J Jr. Glossary of descriptive terminology for ictal semiology: report of the ILAE task force on classification and terminology. Epilepsia (2001) 42:1212–8. 11580774 10.1046/j.1528-1157.2001.22001.x

[33] Beniczky S Neufeld M Diehl B Dobesberger J Trinka E Mameniskiene R. Testing patients during seizures: A European consensus procedure developed by a joint taskforce of the ILAE - Commission on European Affairs and the European Epilepsy Monitoring Unit Association. Epilepsia (2016) 57:1363–8. 10.1111/epi.13472 27440172

[34] Touloumes G Morse E Chen WC Gober L Dente J Lilenbaum R. Human bedside evaluation versus automatic responsiveness testing in epilepsy (ARTiE). Epilepsia (2016) 57:e28–32. 10.1111/epi.13262 26663137 PMC4707993

[35] Koren JP Herta J Fürbass F Pirker S Reiner-Deitemyer V Riederer F. Automated long-term EEG review: fast and precise analysis in critical care patients. Front Neurol. (2018) 9:454. 10.3389/fneur.2018.00454 29973906 PMC6020775

[36] Herta J Koren J Furbass F Hartmann M Kluge T Baumgartner C. Prospective assessment and validation of rhythmic and periodic pattern detection in NeuroTrend: a new approach for screening continuous EEG in the intensive care unit. Epilepsy Behav. (2015) 49:273–9. 10.1016/j.yebeh.2015.04.064 26004320

[37] Hopfengartner R Kasper BS Graf W Gollwitzer S Kreiselmeyer G Stefan H. Automatic seizure detection in long-term scalp EEG using an adaptive thresholding technique: a validation study for clinical routine. Clin Neurophysiol. (2014) 125:1346–52. 10.1016/j.clinph.2013.12.104 24462506

[38] Saab ME Gotman J. A system to detect the onset of epileptic seizures in scalp EEG. Clin Neurophysiol. (2005) 116:427–42. 10.1016/j.clinph.2004.08.004 15661120

[39] Meier R Dittrich H Schulze-Bonhage A Aertsen A. Detecting epileptic seizures in long-term human EEG: a new approach to automatic online and real-time detection and classification of polymorphic seizure patterns. J Clin Neurophysiol. (2008) 25:119–31. 10.1097/WNP.0b013e3181775993 18469727

[40] Arends JBAM. Movement-based seizure detection. Epilepsia (2018) 59(Suppl. 1):30–5. 10.1111/epi.14053 29635767

[41] Hopfengartner R Kerling F Bauer V Stefan H. An efficient, robust and fast method for the offline detection of epileptic seizures in long-term scalp EEG recordings. Clin Neurophysiol. (2007) 118:2332–43. 10.1016/j.clinph.2007.07.017 17889601

[42] Birjandtalab J Baran Pouyan M Cogan D Nourani M Harvey J. Automated seizure detection using limited-channel EEG and non-linear dimension reduction. Comput Biol Med. (2017) 82:49–58. 10.1016/j.compbiomed.2017.01.011 28161592

[43] Furbass F Kampusch S Kaniusas E Koren J Pirker S Hopfengartner R. Automatic multimodal detection for long-term seizure documentation in epilepsy. Clin Neurophysiol. (2017) 128:1466–72. 10.1016/j.clinph.2017.05.013 28622529

[44] Herta J Koren J Furbass F Hartmann M Gruber A Baumgartner C. Reduced electrode arrays for the automated detection of rhythmic and periodic patterns in the intensive care unit: Frequently tried, frequently failed? Clin Neurophysiol. (2017) 128:1524–31. 10.1016/j.clinph.2017.04.012 28501415

[45] McSharry PE He T Smith LA Tarassenko L. Linear and non-linear methods for automatic seizure detection in scalp electro-encephalogram recordings. Med Biol Eng Comput. (2002) 40:447–61. 12227632 10.1007/BF02345078

[46] Zandi AS Dumont GA Javidan M Tafreshi R. Detection of epileptic seizures in scalp electroencephalogram: an automated real-time wavelet-based approach. J Clin Neurophysiol. (2012) 29:1–16. 10.1097/WNP.0b013e318246af3e 22353980

[47] Qu H Gotman J. Improvement in seizure detection performance by automatic adaptation to the EEG of each patient. Electroencephalogr Clin Neurophysiol. (1993) 86:79–87. 7681382 10.1016/0013-4694(93)90079-b

[48] Khamis H Mohamed A Simpson S. Seizure state detection of temporal lobe seizures by autoregressive spectral analysis of scalp EEG. Clin Neurophysiol. (2009) 120:1479–88. 10.1016/j.clinph.2009.05.016 19564130

[49] Minasyan GR Chatten JB Chatten MJ Harner RN. Patient-specific early seizure detection from scalp electroencephalogram. J Clin Neurophysiol. (2010) 27:163–78. 10.1097/WNP.0b013e3181e0a9b6 20461014 PMC2884286

[50] Gotman J. Automatic seizure detection: improvements and evaluation. Electroencephalogr Clin Neurophysiol. (1990) 76:317–24. 10.1016/0013-4694(90)90032-F 1699724

[51] Gabor AJ Leach RR Dowla FU. Automated seizure detection using a self-organizing neural network. Electroencephalogr Clin Neurophysiol. (1996) 99:257–66. 8862115 10.1016/0013-4694(96)96001-0

[52] Gabor AJ. Seizure detection using a self-organizing neural network: validation and comparison with other detection strategies. Electroencephalogr Clin Neurophysiol. (1998) 107:27–32. 9743269 10.1016/s0013-4694(98)00043-1

[53] Wilson SB Scheuer ML Emerson RG Gabor AJ. Seizure detection: evaluation of the Reveal algorithm. Clin Neurophysiol. (2004) 115:2280–91. 10.1016/j.clinph.2004.05.018 15351370

[54] Kuhlmann L Burkitt AN Cook MJ Fuller K Grayden DB Seiderer L. Seizure detection using seizure probability estimation: comparison of features used to detect seizures. Ann Biomed Eng. (2009) 37:2129–45. 10.1007/s10439-009-9755-5 19590961

[55] Schad A Schindler K Schelter B Maiwald T Brandt A Timmer J. Application of a multivariate seizure detection and prediction method to non-invasive and intracranial long-term EEG recordings. Clin Neurophysiol. (2008) 119:197–211. 10.1016/j.clinph.2007.09.130 18037341

[56] Hartmann MM Furbass F Perko H Skupch A Lackmayer K Baumgartner C. EpiScan: online seizure detection for epilepsy monitoring units. Conf Proc IEEE Eng Med Biol Soc. (2011) 2011:6096–9. 10.1109/iembs.2011.6091506 22255730

[57] Furbass F Hartmann M Perko H Skupch A Dollfuss P Gritsch G. Combining time series and frequency domain analysis for a automatic seizure detection. Conf Proc IEEE Eng Med Biol Soc. (2012) 2012:1020–3. 10.1109/embc.2012.6346107 23366068

[58] Baumgartner C Koren JP Automatic seizure detection in epilepsy. Klin Neurophysiol. (2018) 49:8–20. 10.1155/2007/80510

[59] Qu H Gotman J. A seizure warning system for long-term epilepsy monitoring. Neurology (1995) 45:2250–4. 8848202 10.1212/wnl.45.12.2250

[60] Qu H Gotman J. A patient-specific algorithm for the detection of seizure onset in long-term EEG monitoring: possible use as a warning device. IEEE Trans Biomed Eng. (1997) 44:115–22. 10.1109/10.552241 9214791

[61] Shoeb A Edwards H Connolly J Bourgeois B Treves ST Guttag J. Patient-specific seizure onset detection. Epilepsy Behav. (2004) 5:483–98. 10.1016/j.yebeh.2004.05.005 15256184

[62] Jin K Nakasato N. Long-cherished dreams for epileptologists and clinical neurophysiologists: automatic seizure detection in long-term scalp EEG. Clin Neurophysiol. (2014) 125:1289–90. 10.1016/j.clinph.2013.12.105 24439072

[63] Duun-Henriksen J Kjaer T Looney D Atkins M Sørensen J Rose M EEG signal quality of a subcutaneous recording system compared to standard surface electrodes. J Sens. (2015) 2015:1–9. 10.1155/2015/341208

[64] Baldassano SN Brinkmann BH Ung H Blevins T Conrad EC Leyde K. Crowdsourcing seizure detection: algorithm development and validation on human implanted device recordings. Brain (2017) 140:1680–91. 10.1093/brain/awx098 28459961 PMC6075622

[65] Eggleston KS Olin BD Fisher RS. Ictal tachycardia: the head-heart connection. Seizure (2014) 23:496–505. 10.1016/j.seizure.2014.02.012 24698385

[66] Baumgartner C Lurger S Leutmezer F. Autonomic symptoms during epileptic seizures. Epileptic Disord. (2001) 3:103–16. 11679301

[67] Benarroch EE. The central autonomic network: functional organization, dysfunction, and perspective. Mayo Clin Proc. (1993) 68:988–1001. 8412366 10.1016/s0025-6196(12)62272-1

[68] van Elmpt WJ Nijsen TM Griep PA Arends JB. A model of heart rate changes to detect seizures in severe epilepsy. Seizure (2006) 15:366–75. 10.1016/j.seizure.2006.03.005 16828317

[69] Boon P Vonck K van Rijckevorsel K El Tahry R Elger CE Mullatti N. A prospective, multicenter study of cardiac-based seizure detection to activate vagus nerve stimulation. Seizure (2015) 32:52–61. 10.1016/j.seizure.2015.08.011 26552564

[70] Schernthaner C Lindinger G Potzelberger K Zeiler K Baumgartner C. Autonomic epilepsy - the influence of epileptic discharges on heart rate and rhythm. Wien Klin Wochenschr. (1999) 111:392–401. 10413832

[71] Leutmezer F Schernthaner C Lurger S Potzelberger K Baumgartner C. Electrocardiographic changes at the onset of epileptic seizures. Epilepsia (2003) 44:348–54. 12614390 10.1046/j.1528-1157.2003.34702.x

[72] Blumhardt LD Smith PE Owen L. Electrocardiographic accompaniments of temporal lobe epileptic seizures. Lancet (1986) 1:1051–6. 2871334 10.1016/s0140-6736(86)91328-0

[73] Kerem DH Geva AB. Forecasting epilepsy from the heart rate signal. Med Biol Eng Comput. (2005) 43:230–9. 10.1007/BF02345960 15865133

[74] Nei M Ho RT Sperling MR. EKG abnormalities during partial seizures in refractory epilepsy. Epilepsia (2000) 41:542–8. 10.1111/j.1528-1157.2000.tb00207.x 10802759

[75] Galimberti CA Marchioni E Barzizza F Manni R Sartori I Tartara A. Partial epileptic seizures of different origin variably affect cardiac rhythm. Epilepsia (1996) 37:742–7. 8764812 10.1111/j.1528-1157.1996.tb00645.x

[76] Smith PE Howell SJ Owen L Blumhardt LD. Profiles of instant heart rate during partial seizures. Electroencephalogr Clin Neurophysiol. (1989) 72:207–17. 2465123 10.1016/0013-4694(89)90245-9

[77] Massetani R Strata G Galli R Gori S Gneri C Limbruno U. Alteration of cardiac function in patients with temporal lobe epilepsy: different roles of EEG-ECG monitoring and spectral analysis of RR variability. Epilepsia (1997) 38:363–9. 9070600 10.1111/j.1528-1157.1997.tb01129.x

[78] Di Gennaro G Quarato PP Sebastiano F Esposito V Onorati P Grammaldo LG. Ictal heart rate increase precedes EEG discharge in drug-resistant mesial temporal lobe seizures. Clin Neurophysiol. (2004) 115:1169–77. 10.1016/j.clinph.2003.12.016 15066542

[79] Mayer H Benninger F Urak L Plattner B Geldner J Feucht M. EKG abnormalities in children and adolescents with symptomatic temporal lobe epilepsy. Neurology (2004) 63:324–8. 10.1212/01.WNL.0000129830.72973.56 15277628

[80] Surges R Scott CA Walker MC. Enhanced QT shortening and persistent tachycardia after generalized seizures. Neurology (2010) 74:421–6. 10.1212/WNL.0b013e3181ccc706 20124208 PMC2872619

[81] Nilsen KB Haram M Tangedal S Sand T Brodtkorb E. Is elevated pre-ictal heart rate associated with secondary generalization in partial epilepsy? Seizure (2010) 19:291–5. 10.1016/j.seizure.2010.03.003 20395158

[82] Hirsch M Altenmuller DM Schulze-Bonhage A. Latencies from intracranial seizure onset to ictal tachycardia: a comparison to surface EEG patterns and other clinical signs. Epilepsia (2015) 56:1639–47. 10.1111/epi.13117 26282577

[83] Osorio I Schachter S. Extracerebral detection of seizures: a new era in epileptology? Epilepsy Behav. (2011) 22(Suppl. 1):S82–7. 10.1016/j.yebeh.2011.09.012 22078524

[84] Bialer M Johannessen SI Levy RH Perucca E Tomson T White HS. Seizure detection and neuromodulation: a summary of data presented at the XIII conference on new antiepileptic drug and devices (EILAT XIII). Epilepsy Res. (2017) 130:27–36. 10.1016/j.eplepsyres.2017.01.004 28113141

[85] Jeppesen J Beniczky S Johansen P Sidenius P Fuglsang-Frederiksen A. Detection of epileptic seizures with a modified heart rate variability algorithm based on Lorenz plot. Seizure (2015) 24:1–7. 10.1016/j.seizure.2014.11.004 25564311

[86] Conradsen I Beniczky S Hoppe K Wolf P Sorensen HB. Automated algorithm for generalized tonic-clonic epileptic seizure onset detection based on sEMG zero-crossing rate. IEEE Trans Biomed Eng. (2012) 59:579–85. 10.1109/tbme.2011.2178094 22156944

[87] Conradsen I Beniczky S Wolf P Jennum P Sorensen HB. Evaluation of novel algorithm embedded in a wearable sEMG device for seizure detection. Conf Proc IEEE Eng Med Biol Soc. (2012) 2012:2048–51. 10.1109/embc.2012.6346361 23366322

[88] Conradsen I Wolf P Sams T Sorensen HB Beniczky S. Patterns of muscle activation during generalized tonic and tonic-clonic epileptic seizures. Epilepsia (2011) 52:2125–32. 10.1111/j.1528-1167.2011.03286.x 21973264

[89] Larsen SN Conradsen I Beniczky S Sorensen HB. Detection of tonic epileptic seizures based on surface electromyography. Conf Proc IEEE Eng Med Biol Soc. (2014) 2014:942–5. 10.1109/embc.2014.6943747 25570115

[90] Szabo CA Morgan LC Karkar KM Leary LD Lie OV Girouard M. Electromyography-based seizure detector: preliminary results comparing a generalized tonic-clonic seizure detection algorithm to video-EEG recordings. Epilepsia (2015) 56:1432–7. 10.1111/epi.13083 26190150

[91] Halford JJ Sperling MR Nair DR Dlugos DJ Tatum WO Harvey J. Detection of generalized tonic-clonic seizures using surface electromyographic monitoring. Epilepsia (2017) 58:1861–9. 10.1111/epi.13897 28980702 PMC5698770

[92] Beniczky S Conradsen I Henning O Fabricius M Wolf P. Automated real-time detection of tonic-clonic seizures using a wearable EMG device. Neurology (2018) 90:e428–e34. 10.1212/wnl.0000000000004893 29305441 PMC5791791

[93] Beniczky S Conradsen I Pressler R Wolf P. Quantitative analysis of surface electromyography: biomarkers for convulsive seizures. Clin Neurophysiol. (2016) 127:2900–7. 10.1016/j.clinph.2016.04.017 27212115

[94] Beniczky S Conradsen I Moldovan M Jennum P Fabricius M Benedek K. Automated differentiation between epileptic and nonepileptic convulsive seizures. Ann Neurol. (2015) 77:348–51. 10.1002/ana.24338 25545895

